# S-Adenosyl-L-Methionine protects the probiotic yeast, *Saccharomyces boulardii,* from acid-induced cell death

**DOI:** 10.1186/1471-2180-13-35

**Published:** 2013-02-13

**Authors:** Vincent Cascio, Daniel Gittings, Kristen Merloni, Matthew Hurton, David Laprade, Nicanor Austriaco

**Affiliations:** 1 Department of Biology, Providence College, 1 Cunningham Square, Providence, Rhode Island 02918, USA; 2 Present Address: Massachusetts General Hospital, 149 13th St., Rm. 6133, Charlestown, MA 02129, USA; 3 Present Address: Boston University School of Medicine, 72 E Concord St, Boston, MA 02118, USA; 4 Present Address: Tufts University, Miner Hall 222, Medford, MA 02155, USA

**Keywords:** S-Adenosyl-L-Methionine, Saccharomyces boulardii, Probiotic, Acid-induced cell death

## Abstract

**Background:**

*Saccharomyces boulardii* is a probiotic yeast routinely used to prevent and to treat gastrointestinal disorders, including the antibiotic-associated diarrhea caused by *Clostridium difficile* infections. However, only 1-3% of the yeast administered orally is recovered alive in the feces suggesting that this yeast is unable to survive the acidic environment of the gastrointestinal tract.

**Results:**

We provide evidence that suggests that *S. boulardii* undergoes programmed cell death (PCD) in acidic environments, which is accompanied by the generation of reactive oxygen species and the appearance of caspase-like activity. To better understand the mechanism of cell death at the molecular level, we generated microarray gene expression profiles of *S. boulardii* cells cultured in an acidic environment. Significantly, functional annotation revealed that the up-regulated genes were significantly over-represented in cell death pathways Finally, we show that S-adenosyl-L-methionine (AdoMet), a commercially available, FDA-approved dietary supplement, enhances the viability of *S. boulardii* in acidic environments, most likely by preventing programmed cell death.

**Conclusions:**

*In toto,* given the observation that many of the proven health benefits of *S. boulardii* are dependent on cell viability, our data suggests that taking *S. boulardii* and AdoMet together may be a more effective treatment for gastrointestinal disorders than taking the probiotic yeast alone.

## Background

*Saccharomyces boulardii* is a non-pathogenic yeast classified as a probiotic – a live microorganism which, when administered in adequate amounts, confers a health benefit on the host – by the World Health Organization [1]. Available for sale in over 100 countries under the brand name Florastor, this yeast has been prescribed for over fifty years to help maintain the natural flora of the gastrointestinal tract [2,3]. Florastor is also sold as an alternative remedy for acute childhood diarrhea [4] and traveller’s diarrhea [5]. Clinically, *S. boulardii* has been prescribed to treat antibiotic-associated diarrhea (AAD) linked to bacterial infections, especially the AAD associated with *Clostridium difficile*, the cause of about a third of all AAD cases [6-11]. Significantly, the effectiveness of *S. boulardii* as a probiotic has been demonstrated in numerous clinical trials in both pediatric and adult patient populations [9,12-15].

Studies have shown that the therapeutic effect of *Saccharomyces boulardii* can be attributed to the organism’s ability to interfere with and to destroy pathogenic toxins, to decrease bacterial adhesion to intestinal epithelial cells, to preserve the cellular integrity of the intestinal lining, and to restore the intestinal microbiome when it is destroyed [12,16,17]. *S. boulardii* is also able to modify the host’s immune response by either acting as an immune stimulant or by reducing pro-inflammatory responses [18].

Although several studies had suggested that *S. boulardii* is indistinguishable from other strains of *Saccharomyces cerevisiae*, the common baker’s yeast used in laboratories world-wide [3,19,20], more recent work has shown that *S. boulardii* has unique genetic, physiological, and metabolic properties that can be used to differentiate it as a subspecies from *S. cerevisiae*[21,22]. For example, *S. boulardii* grows best at 37°C and is able to tolerate low pH, while *S. cerevisiae* prefers cooler temperatures around 30°C and cannot survive acidic environments [22,23]. These phenotypic differences could explain both why *S. boulardii* can persist in the gnotobiotic mouse models (10d) while *S. cerevisiae* cannot (<1d) [24,25]. Furthermore, the phenotypic differences may also explain why *S. boulardii* can act as a probiotic, while *S. cerevisiae* cannot.

In order to benefit the host, probiotics given orally must not only survive the initial transit through the stomach, but also must be able to persist in the intestine [26]. Studies have reported that only between 1-3% of live yeast is recovered in human feces after oral administration [27,28], as the acidic conditions disrupt cell wall function and cause morphological alterations, leading to cell death [27,29]. However, the nature of this cell death remains unclear.

Recent studies with *Saccharomyces cerevisiae* have shown that this budding yeast is able to undergo programmed cell death (PCD) that is associated with characteristic cell markers reminiscent of apoptosis in mammalian cells including the accumulation of reactive oxygen species (ROS), the condensation of chromatin, the fragmentation of the nucleus, the degradation of DNA, and the activation of caspase-like enzymatic activities [30]. Numerous external stimuli can induce PCD in yeast including hydrogen peroxide, acetic acid, ethanol, high salt, UV irradiation, and heat stress, among others [31-33]. Significantly, one study has shown that *S. cerevisiae* cells undergo apoptotic cell death in acidic environments [34]. PCD has also been linked to intrinsic processes including colony differentiation, replicative and chronological aging, and failed mating events [35-39]. Finally, the process of yeast programmed cell death is mediated by genes that have orthologs that have been implicated in mammalian apoptosis [40].

In this paper we provide evidence that suggests that *Saccharomyces boulardii*, when cultured in either ethanol, acetic acid, or hydrocholoric acid, dies with the fragmentation of mitochondria, the production of reactive oxygen species, and the activation of caspase-like enzymatic activity, three hallmarks of PCD in *Saccharomyces cerevisiae*. Moreover, we show that S-adenosyl-L-methionine (AdoMet), a commercially available dietary supplement, enhances the viability of *S. boulardii* in acidic environments, most likely by preventing programmed cell death. *In toto*, given the observation that many of the proven health benefits of *S. boulardii* are dependent on cell viability, our data suggests that taking *S. boulardii* and AdoMet together may be a more effective treatment for gastrointestinal disorders than taking the probiotic yeast alone.

## Methods

### Yeast strains, plasmids, and growth conditions

All experiments were done with isogenic *Saccharomyces cerevisiae* strains in the W303-1B background (MATα *ade2, his3, leu2, trp1, ura3, ssd1-d2*), and with *Saccharomyces boulardii* (Florastor, Lot No. 538) obtained from Biocodex, Inc. (San Bruno, CA). For all the experiments described in this paper, cells were cultured and treated using standard yeast protocols [41]. Unless noted otherwise, all other drugs and reagents were purchased from SIGMA-Aldrich.

### Ethanol-induced cell death assay

Cells of the indicated strain and genotype were cultured in rich YPD media overnight, resuspended in fresh media, and allowed to reach exponential phase (an approximate OD_600_ value of 0.2). They were then resuspended in water or fresh media or in water or fresh media containing either 15% or 22% ethanol [33], and allowed to grow at 30°C for the indicated times. Next, they were either serially diluted onto YPD plates and cultured at 30°C for 2 days to test for viability or treated with the appropriate stain for the indicated test, and examined using a Zeiss LSM 700 Confocal Laser Scanning Microscope. At least three independent cultures were tested and compared. Statistical significance was determined with the Student’s t-test.

### Acetic acid-induced cell death assay

Cells of the indicated genotype were cultured in rich YPD media overnight, resuspended in fresh media, and allowed to reach exponential phase (an approximate OD_600_ value of 0.2). They were then resuspended in fresh media pH 3 or fresh media pH 3 containing 160mM acetic acid, allowed to grow at 30°C with shaking for 2 hours. Next, they were treated with the appropriate stain for the indicated test, and examined using a Zeiss LSM 700 Confocal Laser Scanning Microscope.

### Hydrochloric acid-induced cell death assay

Cells of the indicated genotype were cultured in rich YPD media overnight, resuspended in fresh media, and allowed to reach exponential phase (an approximate OD_600_ value of 0.2). They were then resuspended in water, water containing either 50 mM or 75 mM HCl, water containing 50 mM HCl and 2 mM AdoMet, or water containing 2 mM AdoMet alone. They were allowed to sit at room temperature for 1.5 hours. Then, they were either serially diluted onto YPD plates and cultured at 30°C for 2 days to test for viability or treated with the appropriate stain for the indicated test, and examined using a Zeiss LSM 700 Confocal Laser Scanning Microscope.

### Viability assay

Cells were grown overnight in rich YPD media at 30°C and then diluted to a final concentration (an approximate OD_600_ value of 0.2). For each strain, a series of 10-fold dilutions was then prepared in water over a range of concentrations from 10^-1^ to 10^-5^ relative to the initial culture. Spots of 5 μl from each dilution series were then plated on the indicated media and cultured at 30°C for 2 days. Individual colonies were then counted and compared to the number of colonies observed from an untreated culture serially diluted at the beginning of the experiment. Several serial dilutions for each culture were done to ensure that there were enough colonies to count for statistical significance and at least three independent cultures were tested and compared. Statistical significance was determined with the Student’s t-test. Note that after 3 hr, cells cultured in rich media without any cell death inducing agents were able to grow and to divide, hence the relative viability levels that are greater than 100%.

### In vivo detection of mitochondrial fragmentation, ROS accumulation, and caspase activation

Mitochondrial fragmentation was detected in *S. boulardii* cells using 10 nM Mitotracker Green (Molecular Probes), according to the manufacturer’s specifications. Intracellular ROS accumulation was examined after treatment with 5 μg/ml of dihydrorhodamine 123 (DHR123; Sigma Aldrich) [42]. Activated caspase-like activity was detected in *S. boulardii* cells after treatment using a FLICA apoptosis detection kit (ImmunoChemistry Technologies, LLC) according to the manufacturer’s specifications [43,44]. After exposure to reagents, *S. boulardii* cells were harvested and examined using a Zeiss LSM 700 Confocal Laser Scanning Microscope.

### Fluorescence microscopy

Cells were grown to mid-log phase in selective media and examined using a 63X oil-immersion objective and a pinhole size of 1 Airy Unit using a Zeiss LSM 700 Laser Confocal Microscope Images were captured and processed using the ZEN 2009 software package.

### Microarray experiments: array design

Genomic sequences were obtained from the *Saccharomyces* Genome Database (downloaded from http://www.yeastgenome.org). These sequences were used to design a custom 8×15K array using the Agilent eArray software (http://earray.chem.agilent.com/). Each array had a minimum of 2 unique 60-mer probes designed against 6,612 open reading frames encoded by *S. cerevisiae*. This resulted in a total of 13,275 unique probes for each array, including Agilent hybridization controls.

### Microarray experiments: sample preparation, extraction, and purification

*S. boulardii* cells were cultured in rich YPD media overnight, resuspended in fresh media, and allowed to reach exponential phase (an approximate OD_600_ value of 0.2). They were then resuspended in 45 mL of either water, for the control condition, or water containing 50 mM HCl for the experimental condition. The total number of cells in each experiment was 3 × 10^8^, as measured with a spectrophotometer. After a 1.5 hr incubation with shaking at room temperature, the cells were washed with 1x PBS, frozen in liquid nitrogen, and stored at −80°C. Total RNA was then extracted using a RiboPure Yeast Kit (Ambion) and purified of gDNA with Turbo DNase (Ambion). RNA was assessed using a NanoDrop-2000c spectrophotometer (Thermo Scientific) and Agilent 2100 bioanalyzer to determine RNA concentration, purity, and integrity.

### Microarray experiments: cDNA synthesis, labeling, and hybridization

cDNA was generated from 10 μg aliquots of purified RNA by first annealing hand-mixed random oligonucleotides (pdN9, 6.3 μg) and oligo(dT)_19_V (8.3 μg) obtained from IDT (Integrated DNA Technologies). First strand cDNA synthesis was then performing using Super Script III reverse transcriptase (Invitrogen) in a reaction containing 0.25 mM DTT and 0.5 mM total deoxynucleoside triphosphates (amino-allyl-dUTP and deoxynucleoside triphosphates) in a ratio of 3:2 aa-dUTP. After synthesis for 3 hr at 42°C, the cDNA was hydrolyzed with 0.3 M NaOH and 0.03 M EDTA. The reaction was then neutralized with 0.3 M HCl to pH 7.0. Following this, cDNA was purified using a 25 ug capacity DNA Concentrator and Cleanup Kit (Zymo), dried using a Speed-vac, resuspended in ddH_2_O (2 μg cDNA per 9 μl water), and stored at −80°C. Dye coupling was achieved by adding 1 μL of 1.0 M NaHCO_3_ solution (pH 9.0) and 1.25 μL of either Cy3 or Cy5 Amersham monoreative dye (GE Healthcare; dissolved in DMSO) to each 9 μL aliquot of cDNA, then incubating for 1 hr at room temperature in darkness. Unincorporated dye was removed and the samples purified using the Zymo cleanup kit. Dye incorporation and cDNA yield were quantified using the NanoDrop-2000c spectrophotometer on the microarray setting. 300 ng of the relevant Cy3- and Cy5-stained cDNAs (control and experiment) were then pooled in a total volume of 25 μL ddH_2_O and denatured at 95°C for 3 min. Following denaturation, 25 μL of 2x HiRPM gene expression and hybridization buffer (Agilent) was added to each sample. These cDNA solutions were then applied to the microarray slide and incubated at 65°C for ~17 hr in a hybridization oven, as per the manufacturer’s instructions. The slides were then sequentially washed in a row of Agilent Wash Buffer I, Agilent Wash Buffer II, and acetonitrile (Sigma), and dried using Agilent drying and stabilization buffer.

### Microarray data analysis and bioinformatics

Slides were scanned using an Axon 4000B scanner (Molecular Devices) and fluorescence was quantified using GENE Pix Pro 3.0 software (Molecular Devices). Data was then normalized using the Goulphar transcriptome platform (http://transcriptome.ens.fr/goulphar/). Duplicate spots for each gene were averaged in Microsoft Excel, and the results were confirmed using qPCR. The Cytoscape 2.8.3 (http://www.cytoscape.org/ download.php) plugin BiNGO 2.44 was used to identify enriched biological processes in differentially expressed genes after Benjamini & Hochberg false discovery correction for multiple hypothesis testing. Pairwise average linkage clustering analysis was performed using the program Cluster and visualized using Treeview [45].

## Results and Discussion

**Figure 1:**
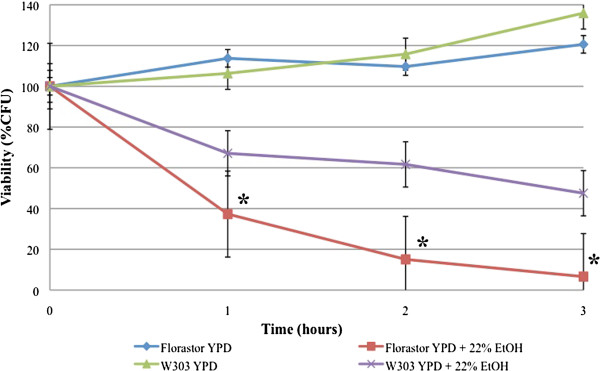
***S. boulardii *has decreased viability in ethanol, similar to *S. cerevisiae.****S. boulardii* (Florastor) and *S. cerevisiae* (W303α) were cultured in rich YPD media overnight and resuspended in fresh media and allowed to reach exponential phase. They were then resuspended in fresh media or in fresh media containing 22% ethanol, allowed to grow at 30°C for the indicated times, serially diluted onto YPD plates, and cultured at 30°C for 2 days. Viability was measured as percentage colony forming units. At least three independent cultures were tested and compared. Note that after 3 hr, cells cultured in rich media without any cell death inducing agents were able to grow and to divide, hence the relative viability levels that are greater than 100%. The differences in viabilities were deemed statistically significant by the Student’s t-test (p<0.05)

*Saccharomyces cerevisiae* cells undergo programmed cell death when they are cultured in media containing either 15% or 22% ethanol [33]. To determine if *S. boulardii* also undergoes PCD, we began by comparing the viabilities of both these strains in ethanol. While the W303α strain shows almost 50% viability after three hours suspended in 22% ethanol, *S. boulardii* shows less than 10% viability after growth in the same media (Figure 1). Our data suggests that *S. boulardii* is less viable in ethanol than this common laboratory strain of *S. cerevisiae*, which is not surprising given the adaptations of brewing yeast, *S. cerevisiae,* that allow it to undergo fermentation efficiently. (Note that after 3 hr, cells cultured in rich media without any cell death inducing agents were able to grow and to divide, hence the relative viability levels that are greater than 100%).

**Figure 2:**
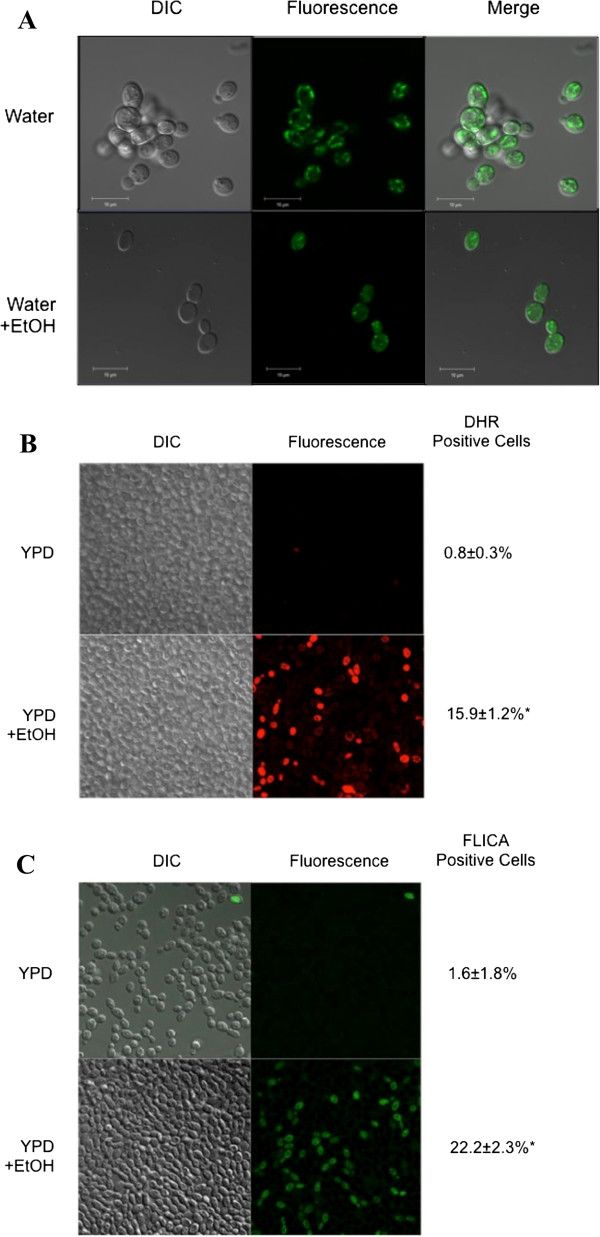
**Like *S. cerevisiae, S. boulardii *cells undergo programmed cell death in ethanol*.****S. Boulardii* cells were cultured in rich YPD media overnight and resuspended in fresh media and allowed to reach exponential phase. They were then resuspended either in water or fresh media or in water or fresh media containing either 15% or 22% ethanol, and allowed to grow at 30°C for the indicated times. (**A**) Mitochondrial fragmentation was detected in cells cultured in 15% ethanol using 10 nM Mitotracker Green. (**B**) Intracellular ROS accumulation was detected in cells cultured in 22% ethanol with 5 μg/ml of dihydrorhodamine 123. (**C**) Activated caspase-like enzymatic activity was detected in *S. boulardii* cells cultured in 22% ethanol using a FLICA apoptosis detection kit according to the manufacturer’s specifications. At least three independent cultures were tested and compared. The differences in staining patterns were deemed statistically significant by the Student’s t-test (p<0.05)

Next, we examined the *S. boulardii* cells dying either in 15% or in 22% ethanol for markers indicative of PCD in yeast, including mitochondrial fragmentation, ROS accumulation, and caspase-like enzyme activation. As shown in Figure 2A, *S. boulardii* cells cultured in 15% ethanol for 1.5 hr had fragmented mitochondria – punctate fluorescence rather than the tubular fluorescence normally seen in wildtype yeast cells – as revealed by MitoTracker Green staining. Cells cultured in ethanol also accumulated ROS (Figure 2B) and manifested a caspase-like activity as measured by a FLICA assay (Figure 2C). Similar findings were obtained with *S. boulardii* cells cultured in 160 mM acetic acid (data not shown), another known inducer of PCD in *S. cerevisiae *[46,47]. Together, these results suggest that *Saccharomyces boulardii,* like *Saccharomyces cerevisiae*, undergoes programmed cell death.

Studies have reported that only between 1-3% of live *S. boulardii* yeast is recovered in human feces after oral administration [27,28] as the acidic conditions disrupt cell wall function and cause morphological alterations that lead to cell death [27,29]. However, the nature of this cell death in acidic environments remains unclear.

**Figure 3:**
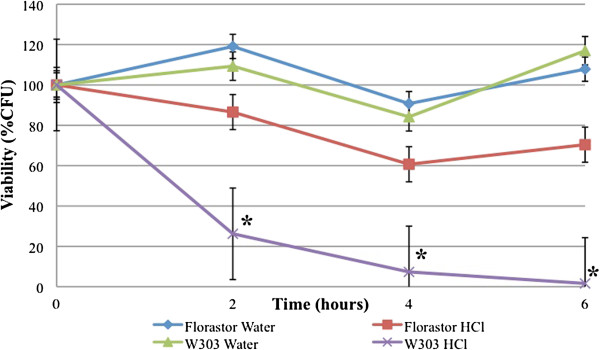
***S. boulardii *cells are more viable in 50 mM HCl than their *S. cerevisiae *counterparts. ***S. boulardii* (Florastor) and *S. cerevisiae* (W303α) were cultured in rich YPD media overnight and resuspended in fresh media and allowed to reach exponential phase. They were then resuspended in water or water containing 50 mM HCl and allowed to grow at room temperature for the indicated times, serially diluted onto YPD plates, and cultured at 30°C for 2 days. At least three independent cultures were tested and compared. The differences in viabilities were deemed statistically significant by the Student’s t-test (p<0.05)

To determine the type of cell death experienced by *S. boulardii* cells in an acidic environment, we began by determining the viability of *S. boulardii* in low pH conditions. Our results show that *S. boulardii* cells have an increased viability in acidic conditions as compared to their *S. cerevisiae* counterpart. After six hours in 50 mM HCl media, W303α cells showed almost no viability, while *S. boulardii* cells were more than 70% viable (Figure 3). This confirms the findings of others who have shown that *S. boulardii* cells are more resistant to acidic conditions than their *S. cerevisiae* cousins [21].

**Figure 4:**
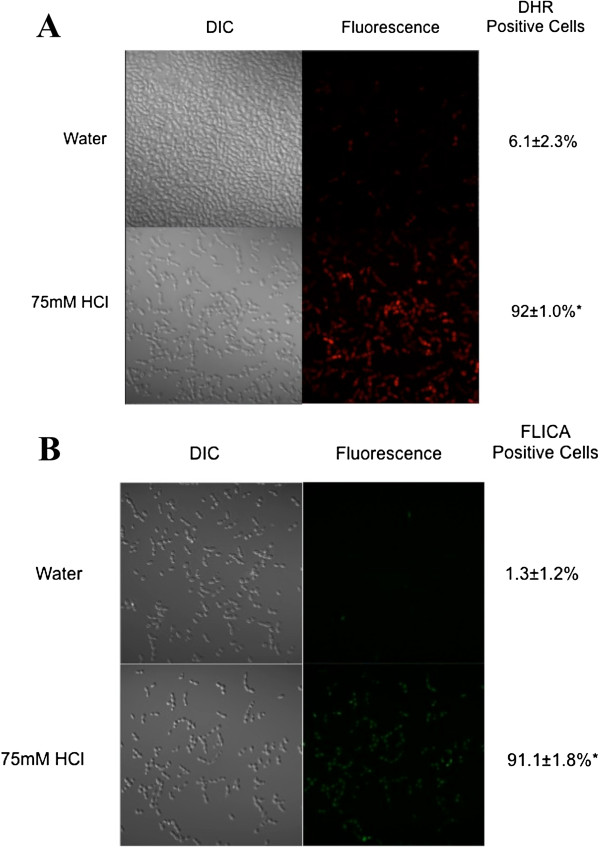
**S. *boulardii *undergoes programmed cell death in an acidic environment. ***S. boulardii* (Florastor) was cultured in rich YPD media overnight and resuspended in fresh media and allowed to reach exponential phase. They were then resuspended in water or water containing 75 mM HCl and allowed to grow at room temperature for 1.5 hr. (**A**) Intracellular ROS accumulation was examined after treatment with 5 μg/ml of dihydrorhodamine 123. (**B**) Activated caspase-like activities were detected using a FLICA apoptosis detection kit according to the manufacturer’s specifications. At least three independent cultures were tested and compared. The differences were deemed statistically significant by the Student’s t-test (p<0.05)

To determine if the *S. boulardii* cells were undergoing PCD in the acidic environment, we repeated our cell death assays with cells cultured in 75 mM HCl (pH 1.5), a scenario that mimics the conditions in the stomach [48]. DHR staining revealed that 92% of the *S. boulardii* cells cultured in an acidic environment contained ROS as compared to cells grown in rich YPD media (Figure 4A). FLICA staining also showed that 90% of the *S. boulardii* cells in the HCl solution, but only 1% of the control cell population had activated caspase-like activity (Figure 4B).

**Figure 5:**
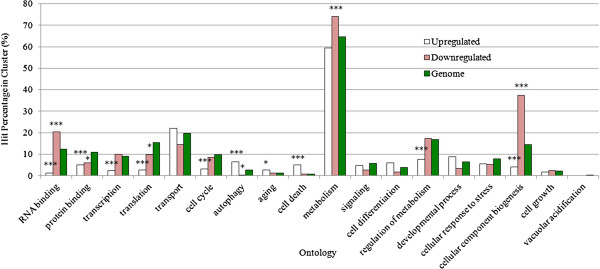
**Functional classification/GO analysis of differentially transcribed genes in *S. boulardii *cells cultured in 50 mM HCl.** Genes showing 2-fold or greater increase (up-regulated) or decrease (down-regulated) in response to an acidic environment were grouped in functional categories. Categories that are significantly enriched relative to the yeast proteome are marked (*: p<0.05; ***: p<0.0005)

Finally, to better understand the mechanism of cell death at the molecular level, we generated microarray gene expression profiles of *S. boulardii* cells cultured in an acidic environment. We found that a total of 947 genes were differentially expressed (log2 values greater than 2 or less that −2) of which 470 were up-regulated and 457 down-regulated (Additional file 1). Significantly, functional annotation revealed that the up-regulated genes were significantly (p<0.0005) over-represented in cell death pathways (Figure 5; Table 1). One of these up-regulated cell death genes, *RNY1*, encodes a RNase T2 family member that is released from the vacuole into the cytosol during oxidative stress to promote yeast cell death [49]. Since the vacuole is the organelle most responsible for pH homeostasis in yeast [50], this may suggest that a similar mechanism of cell death may be occurring in *S. boulardii* cells cultured in an acidic environment. Finally, a significant majority of the other up-regulated cell death genes (80%) were ORFs involved in mitochondrial function, including numerous genes encoding proteins involved in the electron transport chain (Table 1). These microarray results together with our characterization of the cell death phenotype described above suggest that *S. boulardii* cells undergo PCD when they are cultured in acidic conditions similar to those found in the stomach.

**Table 1:** ***S. boulardii *cell death genes differentially expressed in an acidic environment**

**S. BOULARDII CELL DEATH GENES**				
**DIFFERENTIALLY EXPRESSED IN AN ACIDIC ENVIRONMENT**				
**MCD1**		**NMA111**	**NUC1**	**TAH18**
***ATP1***	***ATP2***	***ATP7***	***COR1***	***COX4***
***COX5A***	***COX6***	***COX8***	***CYT1***	***INH1***
***OYE3***	***PIN3***	***POR1***	***QCR2***	***QCR6***
***QCR7***	***QCR8***	***QCR9***	***QCR1O***	***RIP1***
***RNY1***	***SDH1***	***SDH2***	***SDH4***	***UBX6***

*Saccharomyces boulardii* cell death genes showing 2-fold or greater decrease (underlined) or increase (italics) in response to an acidic environment were identified using the Cytoscape 2.8.3 plugin BiNGO 2.44 after Benjamini & Hochberg false discovery correction for multiple hypothesis testing.

**Figure 6:**
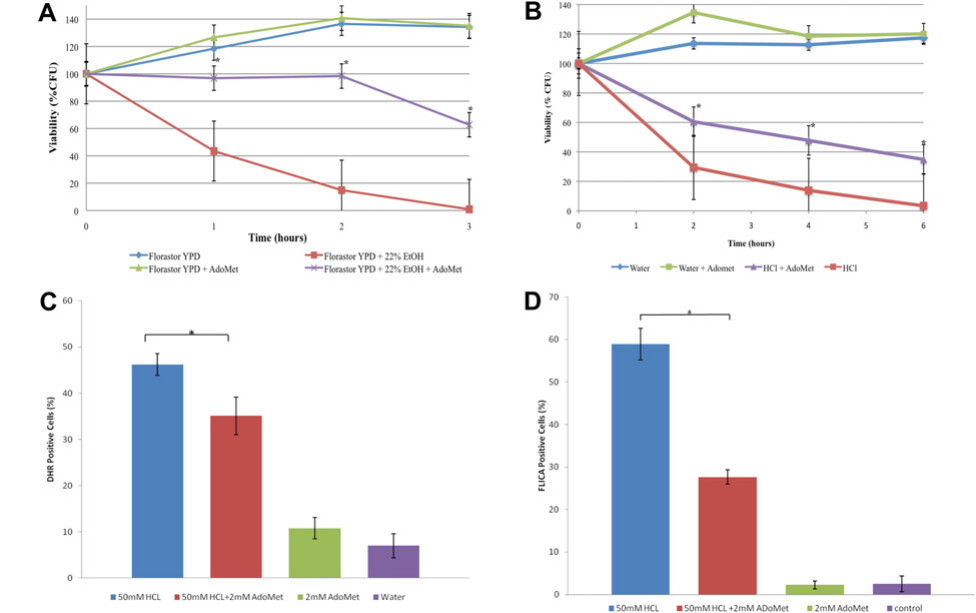
**AdoMet protects *S. boulardii *from ethanol and HCl-Induced cell death. ***S. boulardii* cells (Florastor) were cultured in rich YPD media overnight and resuspended in fresh media and allowed to reach exponential phase. (**A**) They were then resuspended in fresh media, in fresh media containing 22% ethanol, in fresh media containing 1 mM AdoMet, or in in fresh media containing 22% ethanol and 1 mM AdoMet and allowed to grow at 30°C for the indicated times. Viability was measured as percentage colony forming units. (**B**) Next, *S. boulardii* cells were resuspended in water, water containing 75 mM HCl, water containing 75 mM HCl and 2 mM AdoMet, or water containing 2 mM AdoMet alone. They were allowed to grow at room temperature for 1.5 hours. Viability was measured as percentage colony forming units. (**C**) Exponential phase *S. boulardii* cells were resuspended in the indicated culture conditions and allowed to grow at room temperature for 1.5 hr. Intracellular ROS accumulation was detected with 5 μg/ml of dihydrorhodamine 123. (**D**) Activated caspase-like enzymatic activity was detected after treatment using a FLICA apoptosis detection kit according to the manufacturer’s specifications. At least three independent cultures were tested and compared. The differences were deemed statistically significant by the Student’s t-test (p<0.05)

Previously published work has shown that S-adenosyl-L-methionine (AdoMet) protects *S. cerevisiae* from programmed cell death [34]. To determine if AdoMet is also capable of rescuing *S. boulardii* from inducers that induce PCD, we first suspended *S. boulardii* cells either in 22% ethanol or in 22% ethanol containing 1 mM AdoMet, for 3 hours. We discovered that both *S. cerevisiae* and *S. boulardii* cells cultured in ethanol containing AdoMet had higher viabilities than cells cultured in ethanol alone (Figure 6A). These results suggest that AdoMet is also capable of rescuing *S. boulardii* from programmed cell death.

Next, we wanted to determine if AdoMet could also rescue *S. boulardii* cells undergoing HCl-induced programmed cell death. As shown in Figure 6B, the viability of Florastor cells cultured in an acidic environment was significantly enhanced in the presence of 2 mM AdoMet. Next, we showed that 2 mM AdoMet decreased both ROS generation (Figure 6C) and caspase activation (Figure 6D) in *S. boulardii* cells cultured in 50 mM HCl suggesting that this supplement may enhance cell viability by preventing programmed cell death.

## Conclusions

Our study provides evidence that suggests that *S. boulardii* cells undergo programmed cell death in response to stimuli known to induce PCD in *S. cerevisiae*, including an acidic environment. Significantly, we were also able to show that the addition of AdoMet is able to decrease caspase activity and ROS production while increasing viability in *S. boulardii* cells treated with hydrochloric acid. Clinically, these results suggest that taking AdoMet — a commercially available and FDA approved dietary supplement — with *S. boulardii* could be useful in increasing the viability of the yeast during its passage through the acidic environment of the stomach. This should improve its effectiveness both as a probiotic and as a treatment for diarrhea.

## Competing interests

The authors declare no competing interests.

## Authors’ contributions

VC, DG, and KM contributed equally to this paper. Their names are listed in alphabetical order. DL, DG, KM, MH, VC and NA designed, performed, and analyzed the experiments. VC, DL, and NA. wrote the manuscript. All authors read and approved the final manuscript.

## Supplementary Material

Additional file 1 **Differentially Regulated Genes in *S. boulardii *Cells Cultured in an Acidic Environment. ***S. boulardii* genes showing 4-fold or greater increase (up-regulated) or decrease (down-regulated) expression in response to an acidic environment. This data has been submitted to the Gene Expression Omnibus (GEO) at the NCBI with accession number, GSE43271. (XLS 286 kb) Click here for file
